# Next-Generation Sequencing to Determine Changes in the Intestinal Microbiome of Juvenile Sturgeon Hybrid (*Acipenser gueldenstaedtii*♀ × *Acipenser baerii*♂) Resulting from Sodium Butyrate, Β-Glucan and Vitamin Supplementation

**DOI:** 10.3390/genes15101276

**Published:** 2024-09-28

**Authors:** Martyna Arciuch-Rutkowska, Joanna Nowosad, Michał Krzysztof Łuczyński, Syed Makhdoom Hussain, Dariusz Kucharczyk

**Affiliations:** 1 Department of Research and Development, Chemprof, Gutkowo 54B, 11-041 Olsztyn, Poland or martyna.arciuch-rutkowska@uwm.edu.pl (M.A.-R.); michal.luczynski@chemprof.pl (M.K.Ł.); 2 Department of Ichthyology and Aquaculture, University of Warmia and Mazury in Olsztyn, Al. Warszawska 117A, 10-957 Olsztyn, Poland; 3 Department of Ichthyology, Hydrobiology and Aquatic Ecology, National Inland Fisheries Research Institute, Ul. Oczapowskiego 10, 10-719 Olsztyn, Poland; 4 Department of Zoology, Government College University Faisalabad, Punjab 38000, Pakistan; drmakhdoom90@gmail.com

**Keywords:** cortisol level, lysozyme activity, metagenome, short-chain fatty acid, prebiotic, 16S rRNA, α-diversity indexes

## Abstract

Background/Objectives: The effect of sodium butyrate (NaB), β-glucan (βG) and vitamins in the diet on gut microbiome, cortisol level, lysozyme activity and growth parameters of juvenile hybrid sturgeon (*Acipenser gueldenstaedtii*♀ × *Acipenser baerii*♂) was determined. Methods: Sturgeon hybrids (*n* = 144) were divided into three groups with enriched feeding (mg/kg of feed): FQV1 (50 NaB; 20 βG; const. vitamins), FQV2 (150 NaB; 20 βG; const. vitamins), FQV3 (50 NaB; 60 βG; const. vitamins) and control (not supplemented), each group in triplicate, 12 fish in each repetition. Rearing was carried out for 30 days in controlled conditions. Gut microbiome was characterized using Next Generation Sequencing (NGS) of DNA samples isolated from intestinal content. Cortisol level was determined using the ELISA test. Lysozyme activity was measured by turbidimetric test. Results: Based on data obtained from NGS, it was determined that the FQV1 group is characterized by the highest values of diversity indices (Shannon, Simpson and Chao-1) and the largest number of ASVs (Amplicon Sequence Variants). The highest abundance of probiotic bacteria (*Lactobacillus*, *Lactococcus*) was determined in the FQV1 group. The highest cortisol concentration was determined in the control (33.26 ng/mL), while the lowest was in FQV3 (27.75 ng/mL). The highest lysozyme activity was observed in FQV1 (154.64 U/mL), and the lowest in FQV2 (104.39 U/mL) and control (121.37 U/mL) (*p* < 0.05). FQV2 was characterized by significantly more favorable values of breeding indicators (*p* < 0.05). Conclusions: The obtained results prove that an appropriate composition of NaB, βG and vitamins can be used in the commercial breeding of juvenile hybrid sturgeons.

## 1. Introduction

The decline in the number of wild sturgeons, caused mainly by overfishing, poaching and environmental degradation, has indirectly led to the significant development of aquaculture of this group of fish species over recent years [1]. In 1984, sturgeon aquaculture production was 150 metric tonnes, while in the 21st century it increased rapidly and reached approximately 123,500 metric tonnes in 2020 [2]. Currently, sturgeons are farmed all over the world due to obtaining caviar and meat as a secondary product [3]. An important part of sturgeon breeding is their hybrids. In the case of fish, interspecific hybridization is used to improve the breeding characteristics of the offspring, which show better phenotypic characteristics than the parent stocks. One of the most desirable hybrids in sturgeon aquaculture is a hybrid of Siberian and Russian sturgeons. Research conducted by Shivaramu et al. [4] prove a faster growth rate of hybrids of these species and higher survival compared to pure parental lines.

Intensive aquaculture of sturgeons, especially hybrids, has resulted in an increase in demand for specialized feeds that will have a positive impact on their growth and immunity [5]. The introduction of supplementation of basic feeds with active substances can support the immune system in response to infections in the organism of fish, e.g., by increasing the activity of lysozyme as the first line of defense against pathogens (non-specific response) [6,7,8]. Moreover, it is important that these substances are safe for the entire aquatic environment and for humans, as the subsequent consumers [9,10,11]. This group of compounds includes, among others, glucans and salts of short-chain fatty acids (SCFAs), e.g., sodium butyrate [12,13,14,15,16]. These substances support intestinal function and have a prebiotic effect, supporting the nutrition and functioning of the intestinal microbiome of hosts [12,13,17,18].

The intestinal microbiome of fish is still poorly understood, but it is known that, like mammals, it has a significant impact, among others, on host metabolism, reproduction and immunity [19]. The composition of the fish intestinal microbiome is influenced by many factors, such as water temperature and salinity, the host’s eating habits and its trophic level [20]. Through intestinal bacteria, vitamins, enzymes supporting the digestion process and SCFAs, molecules responsible for the nutrition of colonocytes are produced [21]. Additionally, lactic acid bacteria (LAB), by secreting antimicrobial compounds, can fight developing pathogens, leading to the creation of a balanced microbiome community. However, disturbed microflora may also have a negative impact on the growth and development of the host, leading to a decrease in immunity and the development of diseases [21].

The microbiome can be analyzed by traditional microbiological methods and molecular methods. Classic microbiological methods involve many errors, including the fact that only a small percentage of bacteria can be cultivated in laboratory conditions. An effective and quick method for determining the microbiome is the Next-Generation Sequencing (NGS) method [22]. The method involves high-throughput sequencing of the metagenome of an environment based on a marker in the form of the 16S rRNA subunit. The obtained sequences are then assigned to the appropriate species using reference sequence databases [23]. Scientific reports show that the intestinal microbiome is recognized as an “additional organ” and performs many important functions, and that obtaining information on its composition is very important, due to, e.g., nutritional manipulations aimed at improving immunity or growth rate [19,24]. This is particularly important in animal breeding, including aquaculture [24]. It is emphasized that research on the functions of the fish intestinal microbiome may lead to the sustainable development of aquaculture production [23].

The aim of this study was to determine, using the NGS method, the impact of 30-day administration of feed enriched with sodium butyrate, β-glucan and vitamins on changes in the gut microbiome community of juvenile hybrid sturgeon (*Acipenser gueldenstaedtii*♀ × *Acipenser baerii*♂). In addition, the level of cortisol and lysozyme activity in the blood were determined as determinants of immunity. As a part of breeding, indicators reflecting the impact of enriched nutrition on growth were also determined.

## 2. Materials and Methods

### 2.1. The Origin of the Experimental Fish

For the experiment, fish were obtained from the Wąsosze Fishing Farm near Konin (Wielkopolska, Poland). Artificial reproduction was performed according to the protocol described by Fopp-Bayat et al. [25]. The fish were raised to a weight of approx. 5 g and a length of 10 cm. The fry were transported to the Center for Aquaculture and Ecological Engineering (Department of Ichthyology and Aquaculture, Faculty of Animal Bioengineering, UWM in Olsztyn) in bags with oxygen. Before starting the experiment, the fish were acclimatized for a week to adapt to the new conditions. The water temperature was maintained at 18.0 ± 0.1 °C. pH (Hanna HI 98128, Eden Way, UK) and water oxygenation (OxyGuard Pacific, Farum, Denmark) was also monitored. The fish were fed at regular times (8:00, 11:00, 14:00, 16:00) to satiation, with Skretting complete feed (Nutreco; Nutra MP II Protec, Stavanger, Norway), commonly used in Polish farms for raising sturgeon fry with similar body dimensions.

### 2.2. Experimental Feed

The commercial complete feed (Skretting, Nutreco, Nutra MP II Protec, Stavanger, Norway) was used for enrichment with sodium butyrate, β-glucan and vitamin. The detailed composition of the commercial feed is shown in the Table S1. The experimental feed was prepared in three variants: FQV1, FQV2 and FQV3. Feed enrichment was performed according to the methodology described by Nowosad et al. [26] and Arciuch-Rutkowska et al. [27], by introducing 1 mL of an appropriate suspension of active substances (V1, V2, V3) into 50 mL of a mixture of rapeseed oil and water (1:1, *v*:*v*). The whole was homogenized and injected onto 1 kg of commercial feed. The feed for the control group was enriched only with a mixture of oil and water (1:1, *v*:*v*). The enriched feed was mixed and convection-dried for 24 h/23 °C. The content of active substances in the feed after enrichment is presented in Table 1.

### 2.3. Feeding Experiment

A total of 144 juvenile hybrids sturgeons were used to conduct the experiment. At the beginning of the experiment, the average body weight of experimental individuals was 8.56 ± 1.50 g and the average body length was 12.50 ± 0.68 cm (mean ± SD; *n* = 22). Twelve 20 L tanks were used to conduct the experiment and 12 fish were placed in each tank. The experiment was performed for 3 research groups (enriched: FQV1, FQV2, and FQV3) and a control group (not supplemented, C), each in triplicate. The water temperature was kept constant at 18.0 ± 0.1 °C. The following parameters were monitored and noted daily: pH-average 7.8 ± 0.32) (Hanna HI 98128, Eden Way, UK), water oxygenation—average 6.98 ± 0.33 mg/L and saturation—average 84 ± 3.26% (OxyGuard Pacific, Farum, Denmark), nitrite level (LCK 341) and ammonium nitrogen (LCK 304) [28]. During the experiment, the average concentration of nitrite and ammonium nitrogen was below 0.02 mg/L. Fish were fed until satiation four times a day (8:00 a.m., 11:00 a.m., 2:00 p.m., 4:00 p.m.). The experiment was conducted for 30 days. The duration of the experiment was assumed to be until the average body weight of fish in the control group increased by 5 times. The survival rate (SR; %) was calculated for each group at the end of experiment [29].

SR (%) = (N_e_/N_b_) × 100 (1)

N_b_—initial number of fish; N_e_—final number of fish

### 2.4. Periodic Measurement of Fish and Sampling

Measurements of body weight and length of experimental individuals were performed weekly during the experiment and at the end of the experiment. Fish were caught from each group (*n* = 15) and anesthetized using MS-222 anesthetic (Sigma-Aldrich, Saint Louis, MO, USA) (0.15 g/L water). Fish were weighed (±0.01 g; KERN ABJ; 320-4NM, Frankfurt, Germany) and measured using a caliper (±0.1 mm; Geko G01493, Kietlin, Poland). After completing all the data from the experiment, they were used to calculate breeding indicators: weight gain (WG; g), length gain (LG; cm), specific growth rate (SGR; % d^−1^), and condition factor (K; g/cm^3^), according to the formulas [30,31]:

WG (g) = (FW − IW)(2)

FW—final weight (g), IW—initial weight (g)

LG (cm) = (FL − IL)(3)

FL—final length (cm), IL—initial length (cm)

SGR (%) = (lnFW − lnIW)/T × 100%(4)

FW—final weight (g), IW—initial weight (g), T—number of days of rearing

K (g/cm^3^) = (BW·100)·L^−3^ (5)

BW—body weight (g), L—body length (cm)

In order to collect samples for further analysis, after the end of the experiment, randomly selected fish (*n* = 5 from each group) were euthanized using an overdose of MS-222 (Sigma–Aldrich, Saint Louis, MO, USA). Blood was collected from the tail vein into heparin tubes (FL Medical, Torreglia, Italy). To obtain plasma, the blood was centrifuged (10,000× *g*/10 min; IKA G-L S000, IKA, Staufen, Germany) and frozen until cortisol concentration and lysozyme activity analyses were performed [32]. The surface of the fish body was disinfected (70% isopropanol) and the contents of the intestines were collected into sterile tubes under sterile conditions (ESCO AC2-3E8-TU, Singapore). Samples were stored at −80 °C until metagenomic analyzes began [33].

### 2.5. DNA Isolation, NGS Sequencing and Bioinformatic Analysis

DNA from the intestine contents was isolated using the commercial QIAamp Fast DNA Stool Mini Kit (Cat. no. 51604; Qiagen, Hilden, Germany). The manufacturer’s protocol was used for the DNA isolation. After isolation, the quality of DNA was examined by electrophoresis in agarose gel. The quantity and purity of the DNA samples were determined using a Nanodrop spectrophotometer (Thermo Scientific Nanodrop One, Waltham, MA, USA). Samples of metagenomic DNA were stored at −80 °C. NGS (Next-Generation Sequencing) was performed at an external company (Genomed SA; Warsaw, Poland) using the MiSeq system (Illumina, San Diego, CA, USA) and paired-end (PE) technology (2 × 300 nt) using v3 kit (Cat. no. MS-102-3003; Illumina, San Diego, CA, USA). A protocol 16S Metagenomic Sequencing Library Preparation (Illumina, San Diego, CA, USA) and specific primers 341F and 785R were used to prepare the libraries. Metagenomic analysis was performed based on the 16S rRNA gene (V3–V4 region). Q5 Hot Start High-Fidelity 2X Master Mix (Cat. no. M0494X; New England Biolabs, Ipswich, MA, USA) was used for PCR following the manufacturer’s protocol.

QIIME 2 was used to perform bioinformatics processing [34]. Raw reads were classified to the taxon level based on the generated ASVs and the Silva 138 reference sequence database. The DADA2 package was used to distinguish sequences of biological origin from sequences generated during sequencing [35]. The FIGARO tool was used to check the quality of readings. Adapter sequences and short reads were removed during the analyses [36].

### 2.6. Analysis of Cortisol Concentration and Lysozyme Activity in the Blood Plasma

The commercial Fish (Cortisol) ELISA Kit (Cat. no. 201-00-0027; SunRed Biological Technology, Shanghai, China) was used for analysis. Cortisol concentration analysis was performed according to the manufacturer’s procedure instructions, using a microplate reader (BK-EL10C, BIOBASE, Shandong, China) at a wavelength of 450 nm. The test’s detection range was set at 0.8–200 ng/mL, with a minimum sensitivity of 0.712 ng/mL.

Lysozyme activity in blood plasma was determined using the turbidimetric method using the commercial Lysozyme Activity Kit (Cat. no. LY0100; Sigma-Aldrich, Saint Louis, MO, USA). The assay was performed according to the manufacturer’s protocol, which involved the lysis of *Micrococcus lysodeikticus* cells. The absorbance of the tested samples was measured at 450 nm at 25 °C using a UV/Vis spectrophotometer (Lambda 265, PerkinElmer, Waltham, MA, USA). The measurement was performed in two repetitions against a blank sample. The test’s detection limit was 0.8 mg/mL. Lysozyme activity (LA; U/mL) was calculated from the formula [37]:

LA (U/mL) = (ΔA 450/min test − ΔA 450/min blank) df/(0.001)(V test)(6)

df—dilution factor; 0.001—ΔA _450_ (as per the unit definition); V _test_—volume of sample (mL)

One unit of lysozyme leads to a change of 0.001 in the ΔA _450_ value per minute at pH 6.24 at 25 °C, using a suspension of *M. lysodeikticus* as substrate in 2.6 mL of the reaction mixture.

### 2.7. Statistical Analysis

Statistica 13.3 (TIBCO Software Inc.; Palo Alto, CA, USA) was used to perform statistical analyses. Shapiro–Wilk and Leven tests were used to verify normality and homogeneity of variances. Parameters such as WG, LG, SGR, SR, cortisol concentration and lysozyme activity in the blood were statistically analyzed using one-way ANOVA and Tukey’s test as a post hoc test (*p* < 0.05).

The results of the bioinformatic analysis are given as the mean with standard deviation. The α-diversity were calculated using the PAST 4.03 program (University of Oslo, Oslo, Norway). Statistical analysis was performed using the non-parametric Kruskal–Wallis test (*p* < 0.05). β-diversity was plotted using PCoA (Principal Coordinate Analysis) with the Bray–Curtis distance (PAST4.03). Relative abundance of bacteria (%) was calculated at the Phylum and Genus levels, and presented in bar charts.

## 3. Results

### 3.1. Breeding Indicators

Breeding indicators of juvenile sturgeon hybrids are presented in Table 2. The highest WG value was observed in the FQV2 group (47.24 ± 10.54 g), while the lowest was in the FQV3 group (39.66 ± 8.68 g) (*p* < 0.05). The FQV2 group also had the highest LG (10.57 ± 1.31 cm) and SGR (4.82 ± 0.67) (*p* < 0.05). The lowest SGR value was demonstrated in the FQV3 group (4.38 ± 0.57%). The condition factor (K) and SR were at a similar level in all groups and no significant statistical differences were found between the groups (*p* > 0.05).

### 3.2. Sequencing Data and Diversity of Gut Microbiome

Data from metagenomic sequencing of DNA isolated from the intestinal contents of the experimental fish are summarized in Table 3. Bioinformatics analyses allowed for the taxonomic classification of 2,008,080.00 reads. On average, there were 100,404.00 ± 23,546.31 readings per sample. A total of 1785.00 ASV sequences were also extracted from all analyzed samples. The number of counts in all groups was at a similar level, but the highest was in the FQV2 group (107,145.60 ± 37,173.80). Differences between groups were not statistically significant (*p* > 0.05). In the FQV1 group, the number of observations was the highest (254.80 ± 46.91), while in groups C, FQV2 and FQV3, the number of observations was at a similar level. There were no statistically significant differences between the study groups (*p* > 0.05).

**Figure 1:**
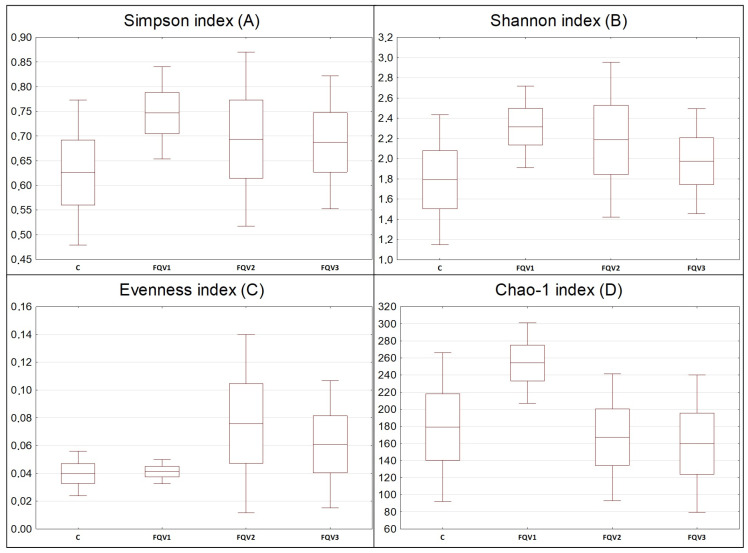
α-diversity indexes of intestinal microbiome juvenile sturgeon hybrid (*Acipenser gueldenstaedtii* × *Acipenser baerii*) fed commercial feed in the control group (C) and enriched feed with sodium butyrate, β-glucan and vitamins in research groups (FQV1, FQV2, and FQV3) during a feeding experiment.

α-diversity coefficients (Simpson index, Shannon index and Evenness index) and Chao-1 index was determined for the studied groups (Figure 1). The FQV1 group was characterized by the highest Simpson index (0.75 ± 0.09) and Shannon index (2.32 ± 0.40). In the control group, the above-mentioned indexes had the lowest values: for the Simpson index it was 0.63 ± 0.17, and for the Shannon index it was 1.79 ± 0.64. Evenness index had the highest value in the FQV2 group (0.08 ± 0.06), while the lowest was in the control group and FQV1; in both cases, its value was 0.04 ± 0.01. Index values did not show statistically significant differences between groups (*p* > 0.05). The FQV1 group was characterized by the highest Chao-1 index (254.20 ± 46.95), while in the FQV3 group this index had the lowest value of all groups (159.80 ± 80.29). Differences in the Chao-1 index value between groups were not statistically significant (*p* > 0.05).

**Figure 2:**
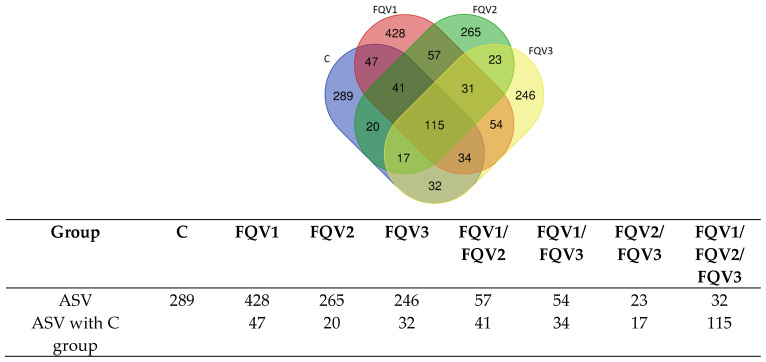
Common and unique ASVs of the intestinal microbiome of juvenile sturgeon hybrid (*Acipenser gueldenstaedtii* × *Acipenser baerii*) fed commercial feed in the control group (C) and enriched feed with sodium butyrate, β-glucan and vitamins in research groups (FQV1, FQV2, and FQV3) during a feeding experiment.

The number of common and unique ASVs is shown in a Venn diagram and table (Figure 2). The analysis showed that the number of common ASVs occurring in all groups was 115. However, the group with the highest rate of unique ASVs was the FQV1 group (428). The FQV3 group had the lowest number of unique ASVs (246).

**Figure 3:**
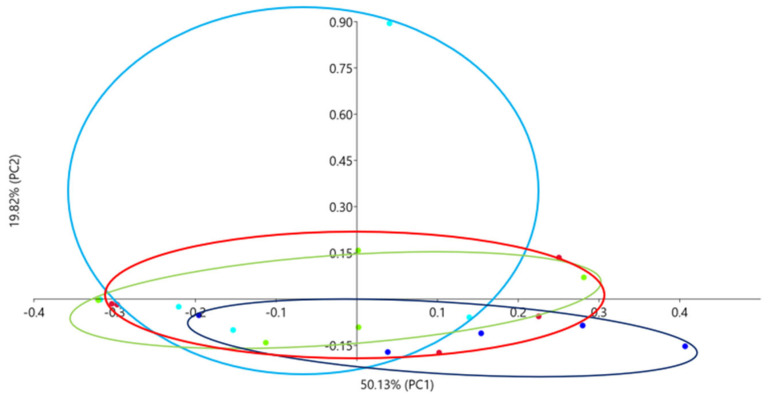
PCoA (Principal Coordinate Analysis) analysis of the intestinal microbiome of juvenile sturgeon hybrid (*Acipenser gueldenstaedtii* × *Acipenser baerii*) fed commercial feed in the control group (C) and enriched feed with sodium butyrate, β-glucan and vitamins in supplemented groups (FQV1, FQV2, and FQV3) during a feeding experiment. Points of the same color mean individuals from the same group (C—light blue points, FQV1—dark blue points, FQV2—red points and FQV3—green points).

The β-diversity of the microbiome community was analyzed by PCoA analysis using the Bray–Curtis distance. The results of the analysis are presented at Figure 3. The research groups (FQV1–FQV3) show great similarity in terms of microbiome community, with the FQV2 and FQV3 groups being the most similar. The control group was the group with the greatest microbiome diversity.

### 3.3. Profile of Gut Microbiome

**Figure 4:**
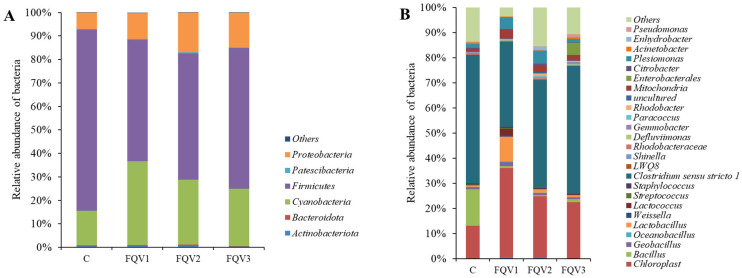
Relative abundance of bacteria (%) at Phylum (**A**) and Genus (**B**) level in the intestinal microbiome of juvenile sturgeon hybrid (*Acipenser gueldenstaedtii* × *Acipenser baerii*) fed commercial feed in the control group (C) and enriched feed with sodium butyrate, β-glucan and vitamins in research groups (FQV1, FQV2, and FQV3) during a feeding experiment. Bacteria less than 0.5% are summarized and labeled as “others”.

Based on data obtained from bioinformatic analyses, the profile of intestinal microbiome of juvenile sturgeon hybrids was determined in the research groups (FQV1, FQV2, and FQV3) and in the control group (C). At the Phylum level in all research groups and the control group, *Firmicutes* dominated, with the largest share in the control group (77.25%; Figure 4A). In the research groups (FQV1–FQV3), the share of *Firmicutes* was over 50%. *Cyanobacteria* were present in all groups, most abundantly in the FQV1 (35.51%), and least frequently in the control group (14.69%). *Proteobacteria* had a large share in the microbiome community of all groups. The control group showed the lowest occurrence (6.89%), and in the research groups *Proteobacteria* occurred at the level of approximately 15%.

The graph (Figure 4B) shows the relative abundance of bacteria of all groups at the Genus level. The most abundant type of bacteria in all groups is *Clostridium sensu stricto 1*. It is the most abundant bacteria in the control group—57.74%, while in the research groups, respectively, FQV1—33.90%, FQV2—48.16%, and FQV3—55.23%. In the control group, *Bacillus* has a large share (16.35%), while in the research groups their share ranges from 0.58% (FQV2) to 1.36% (FQV3). Lactic acid bacteria (LAB), *Lactobacillus* and *Lactococcus* are most abundant in the FQV1 group, respectively, *Lactobacillus*—9.82%, *Lactococcus*—2.52%. In the remaining groups, the percentage share in the microbiome community was approximately 1% or less. Additionally, *Plesiomonas* was also present, most often in the FQV2 (5.88%). In the control and FQV3 groups, the share of Plesiomonas was below 2%. *Shinella* was statistically most common in the FQV3 group, and least frequent in the FQV1 group (*p* < 0.05). However, the occurrence of *Rhodobacter* statistically differed between the groups; it was most common in the FQV2 group and least common in the control group.

### 3.4. Cortisol Concentration and Lysozyme Activity in Blood Plasma

**Figure 5:**
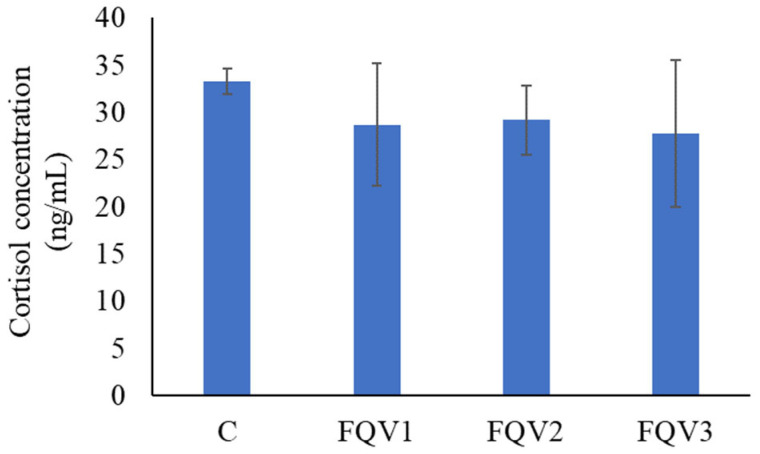
Cortisol concentration (ng/mL) in the blood plasma (mean ± SD) of juvenile sturgeon hybrid (*Acipenser gueldenstaedtii* × *Acipenser baerii*) fed commercial feed in the control group (C) and enriched feed with sodium butyrate, β-glucan and vitamins in research groups (FQV1, FQV2, and FQV3) during a feeding experiment.

The control group had the highest cortisol concentration (33.26 ± 1.30 ng/mL) compared to the research groups, but the differences between the groups were not statistically significant (*p* > 0.05). The lowest cortisol level was observed in the FQV3 group (27.75 ± 7.80 mg/mL) (Figure 5).

**Figure 6:**
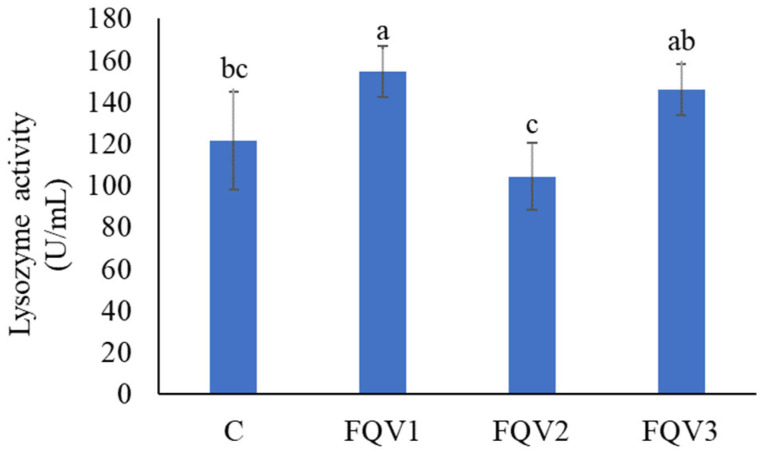
Lysozyme activity (U/mL) in blood plasma (mean ± SD) of juvenile sturgeon hybrid (*Acipenser gueldenstaedtii* × *Acipenser baerii*) fed commercial feed in the control group (C) and enriched feed with sodium butyrate, β-glucan and vitamins in research groups (FQV1, FQV2, and FQV3) during a feeding experiment. Bars marked with a different letter are statistically different (*p* < 0.05).

The highest lysozyme activity was observed in FQV1 (154.64 ± 12.01 U/mL) (*p* < 0.05). In the FQV2 group, the lowest enzymatic activity in blood plasma was determined, and was 104.39 ± 15.94 U/mL, respectively, (*p* < 0.05) (Figure 6).

## 4. Discussion

This study used the NGS metagenomic sequencing method to determine changes in the diversity and profile of gut microbiome of sturgeon hybrid (*Acipenser gueldenstaedtii♀* × *Acipenser baerii♂*) as a result of enriching the diet with sodium butyrate, β-glucan and vitamins compared to the control group fed with unenriched feed. The FQV1 group was characterized by the highest values of α-diversity and species richness indices. Moreover, in the FQV1 group, the highest percentage of probiotic bacteria was determined, i.e., *Lactobacillus* and *Lactococcus*, compared to the other groups. Other parameters indicating the level of immunity were also checked, i.e., cortisol level and lysozyme activity in the blood. The introduced supplementation did not cause a significant reduction in the level of cortisol in the blood of the tested fish. Compared to the control group, the supplemented groups recorded an average decrease in blood cortisol concentration of 14.22%. The highest lysozyme activity in the blood was determined in the FQV1 group, which was on average 27.41% higher than in the control group. The influence of enrichment on the growth rate of the tested fish was also determined.

Intestinal microbiome is one of the main factors that have a direct impact on the health and immunity of animals. Compared to mammals, the gut microbiome of fish is poorly understood. Nevertheless, it is known that the composition of the microbiome depends on the fish species, environmental conditions and, above all, diet [38]. In recent years, NGS metagenomic sequencing has been widely used in research on the fish microbiome community, due to the accuracy of the data obtained and relatively low cost [23]. Present study investigated the impact of sodium butyrate and β-glucan addition on the biodiversity and gut microbiome profile of sturgeon using NGS data. Numerous scientific works emphasize the importance of the biodiversity of the microbiome community in the fight against pathogens and inflammation [39,40,41]. The introduction of sodium butyrate and β-glucan supplementation to the diet of African catfish at the fry and juvenile stages caused modulation of α-diversity indicators such as Shannon, Simpson and Chao-1 index, compared to the unsupplemented control group [27,42]. This is because the active substances such as sodium butyrate and β-glucan have a direct and indirect effect on the intestinal microbiome and its biodiversity [42]. β-glucan is a source of energy and a nutrient for intestinal bacteria, influencing their growth in the intestinal environment [43]. In contrast, sodium butyrate increases the growth of bacteria producing pro-inflammatory SCFAs [44]. This study showed that dietary supplementation of juvenile hybrid sturgeon in the FQV1 group significantly influenced the α-diversity indices of the intestinal microbiome compared to the control group. The Chao-1 index indicating the species richness of the fish microbiome in the FQV1 group is approximately 1.4 times higher than in the control group. The above is also confirmed by the analysis of unique ASVs, which indicate species diversity. A significant increase in the number of unique ASVs was observed in the FQV1 group compared to the other groups and the control group (approximately 1.5x). The results presented by Bozzi et al. [45] prove that the high species diversity of the microbiome allows for the cooperation of microorganisms in the intestinal environment, especially in the event of inflammation in the body. This statement is also confirmed in this paper. In the FQV1 group, where the highest species biodiversity was observed, the highest activity of lysozyme was also determined, which is responsible for one of the first lines of defense against pathogens [46].

Increase in the relative number of LAB bacteria of the genus *Lactobacillus* and *Lactococcus* was observed in the FQV1 group compared to the other groups. In the control group, the highest share of pathogenic bacteria—*C. sensu stricto 1*—was determined, while the FQV1 group had the lowest share. This bacterium is an opportunistic pathogen, responsible for the occurrence of intestinal inflammation [47]. Other authors also reach similar conclusions, observing an increased frequency in LAB occurrence and a simultaneous reduction in the number of pathogenic bacteria due to the supplementation of the fish diet with salts of organic acids and prebiotics. Tian et al. [48] showed that enriching the diet of juvenile grass carp (*Ctenopharyngodon idella*) with sodium butyrate has a positive effect on the intestinal microbiome profile, promoting the *Lactobacillus* genus, while reducing the number of pathogenic bacteria, i.e., *Aeromonas* and *Escherichia coli*. Adbel-Mohsen et al. [18], in their research, also confirmed the positive effect of sodium butyrate on the microbiome of European seabass fry (*Dicentrachus labrax*). β-glucan also modulates the composition of the fish gut microbiome. An example is research conducted on Senegalese sole (*Solea senegalensis)*, where β-glucan obtained from yeast was used as a dietary supplement. It has been shown that β-glucan administered with the diet has a positive effect in reducing the number of pathogenic bacteria *Vibrio sp* [49].

In the group fed with the feed with the highest content of β-glucan-FQV3 (60 mg/kg) and sodium butyrate at the level of 50 mg/kg, a high level of lysozyme activity in the blood was observed, compared to the control group. It turns out that neutrophils, Natural Killer (NK) cells and macrophages have glucan receptors, the presence of which activates cells and immune response mechanisms, i.e., phagocytosis and cytokine release. These cytokines stimulate the production of new white blood cells, which increase the secretion of lysozyme [50]. Studies conducted on yellow croaker (*Pseudosciaena crocea*), Indian carp (*Labeo rohita*) and sea bream (*Pagrus major*) also prove that enriching the diet with β-glucan increases the activity of lysozyme in the blood, compared to unsupplemented groups [51,52,53]. Moreover, it is found that β-glucan in the diet contributes to increased resistance to stress caused by low salinity [53]. A commonly accepted measure of stress in fish is the level of cortisol, a glucocorticoid hormone released into the blood by intrarenal cells. As a result of long-term stress and, consequently, high levels of cortisol in the blood, immunosuppression may occur. This condition contributes to the impairment of the immune response, which makes the fish more vulnerable to bacterial or viral infections [54]. It turns out that supplementing the fish diet with β-glucan contributes to the inhibition of cortisol secretion. Lopes et al. [13], in their studies on pacu (*Piaractus mesopotamicus*), also confirm that β-glucan significantly reduces the concentration of cortisol in the blood, even during bacterial infection with *Aeromonas hydrophila*. Similar conclusions were also reached by Dawood et al. [55], examining the effect of β-glucan in the diet on, among others, blood cortisol levels after exposure to chlorpyrifos. The use of Nile tilapia β-glucan in the diet significantly reduced the level of cortisol in the blood, compared to the unsupplemented group. The influence of 0.1% and 0.5% β-glucan solution administered in the form of a bath on the level of cortisol in the blood and the healing of wounds in silver catfish (*Rhamdia quelen*) was also investigated. It was found that daily bathing in 0.5% β-glucan reduces the level of cortisol in the blood and accelerates wound healing [56]. However, in this study, no significant reduction in cortisol in the blood was observed after using b-glucan as a dietary supplement.

Many scientists focus on supplementing animal feed with single active ingredients such as sodium butyrate or β-glucan. Sodium butyrate is a particularly interesting compound, due to its proven effect on stimulating the growth and improving the intestinal function also of aquatic animals [57]. Studies on the feeding of largemouth bass (*Micropterus salmoides*) have shown that the higher the dose of butyrate in the diet (up to 2 g/kg of feed), the more favorable the growth parameters such as body weight gain rate (WGR) and SGR [58]. Research conducted by Fang et al. [59] on Pengze Prussian carp (*Carassius auratus var pengze*) show a similar trend. The dose of sodium butyrate at the level of 2.0–4.0 g/kg of feed caused a faster increase in fish weight compared to the group fed with feed with a lower butyrate content (1.0 g/kg) and the control group. Studies on dietary supplementation of juvenile golden pompano (*Trachinotus ovatus*) with sodium butyrate showed a similar relationship, finding an improvement in final body weight and SGR with increasing sodium butyrate content in the diet (up to 2.0 g/kg feed) [60]. Studies conducted on arapaima fish (*Arapaima gigas*) indicate that the most beneficial dose is 1.2 g of butyrate/kg of feed. This dose induces the best weight gain and SGR, and also improves SR [61]. The literature review and the presented data show that the optimal doses of sodium butyrate in the diet as a growth stimulator largely depend not only on the form, but also on the species, of the fish examined.

Many studies also focus on single supplementation of dietary β-glucan on breeding parameters and parameters of the immune response of aquatic animals. The effect of dietary supplementation of juvenile Persian sturgeon (*Acipenser persicus*) with β-glucan in the form of the commercial product MacroGard was examined. It has been shown that a dose of β-glucan of 2.0 g/kg of feed causes an acceleration in the growth rate and SGR of the tested fish [62]. Similar conclusions were also reached by Do Huu et al. [63], examining the effect of β-glucan on the breeding performance of pompano fish (*T. ovatus*). The dose of β-glucan at the level of 0.1–0.2% significantly improved the final body weight and SGR, and also increased the survival rate of the fish. A dietary dose of 0.2% β-glucan was also used by Khanjani et al. [64], examining its effect on the growth of rainbow trout (*Oncorhynchus mykiss*). A significant improvement in the survival rate and growth rate of fish after a period of enriched feeding was demonstrated. However, research conducted on Nile tilapia (*Oreochromis niloticus*) indicates that dietary supplementation with β-glucan has no effect on growth rate [65]. However, it was observed that a dose of 0.4–0.5% significantly promoted the immune response and resistance to infections in the tested fish. Sealey et al. [66], in their studies on rainbow trout, found a positive effect on survival during IHNV viral infection. β-glucan in combination with other ingredients, such as mannan oligosaccharide and *Lactobacillus plantarum*, produces much better effects than each of these ingredients given separately in the fish diet. Their synergistic effect improves growth parameters, as well as the innate immune response [67].

The beneficial doses of sodium butyrate and β-glucan in the fish diet as growth stimulants largely depend on the tested fish species and/or additional substances with a synergistic effect. In this study, the most favorable values of breeding indicators were determined in the group fed with feed enriched with the FQV2 variant, which contained the highest amount of sodium butyrate-150 mg/kg of feed among all research groups and β-glucan, at the level of 20 mg/kg of feed. Appropriate feed utilization by farmed fish indirectly affects the growth rate, and is associated with the economic effect of breeding. However, the intestinal microbiome of fish is crucial in improving the bioavailability of nutrients contained in feed. Studies on determining the influence of intestinal microorganisms on feed utilization were mainly conducted on mammals. There is little information in the literature on the role of the fish intestinal microbiome in feed absorption [68,69]. In fish, the mechanisms responsible for the regulation of metabolism by the intestinal microbiome are insufficiently understood. It is assumed that the mechanisms of SCFA formation as secondary metabolites of the intestinal microbiome in fish and mammals are similar. However, the effects of SCFAs on fish metabolism through appropriate receptors require further research [38].

β-glucan, used by the intestinal microbiome as an energy source, is converted to SCFA, mainly to acetate, propionic and butyric acid. However, as a result of butyrate dissociation, butyric acid is formed [70]. The resulting SCFAs are mediators between intestinal microorganisms and the host. SCFAs, especially butyric acid, play a significant role in the nutrition of colonocytes, enabling their regeneration and proliferation by increasing the height of the intestinal villi [71]. The result is better absorption and utilization of nutrients contained in the feed in metabolic pathways [10,72,73]. SCFAs also improve the bioavailability of certain dietary amino acids by preventing their oxidation in the gastrointestinal tract [74]. In addition, SCFAs stimulate the exocrine function of the pancreas by increasing the secretion of digestive enzymes. These enzymes contribute to improving the digestibility and assimilability of feed [75]. Moreover, it turns out that the composition of the intestinal microbiome of predatory fish species such as sturgeon is closely related to the activity of the protein-digesting enzyme trypsin, and, in herbivorous species, to the activity of cellulase and amylase [76]. Short-chain fatty acids are also involved in maintaining an appropriate profile of intestinal microorganisms by regulating the secretion of antimicrobial proteins by the intestinal epithelium [77]. It turns out that SCFAs produced in the intestines, after entering the bloodstream, also affect other organs. As a result of connection with appropriate receptors on the surface, they regulate many processes, thus influencing metabolism, inflammation and immunity [78]. There is little scientific information about the role of SCFAs on the fish, but research conducted on zebrafish (*Danio rerio*) suggests that butyric acid derived from the fermentation of polysaccharides has an impact on the regulation of resistance to infections, increasing the expression of IL-1β and the percentage of intestinal neutrophils [79].

Many scientists undertake research to determine the appropriate time for administering supplements, in order to reduce it to a minimum. It turns out many times that a monthly (or even shorter) supplementation period is sufficient to notice significant changes in the intestinal microbiome, immunity or growth. This finding is consistent with studies conducted on other fish species. A four-week period of rearing juvenile African catfish (*Clarias gariepinus*) using the above-mentioned substances was sufficient to notice significant changes in the intestinal microbiome profile—an increase in the *Lactobacillus* population and a decrease in the percentage of the pathogenic *Candidatus* Arthromitus strain, compared to the control group. Moreover, a significant improvement in growth rate was observed in all supplemented groups, together with a reduction in cannibalism [27]. Another research on juvenile African catfish using bee pollen as a feed additive at various concentrations has shown that a three-week rearing period with enriched nutrition is sufficient to significantly improve growth parameters and induce positive changes in the intestinal microbiome, leading to an increase in lactic acid bacteria, compared to the control group [26]. Similarly, research on juvenile *P. mesopotamicus* demonstrates that even a 14-day period of feeding with β-glucan-enriched feed is enough to produce noticeable effects, such as reduced cortisol levels both at rest and after bacterial infection, thereby confirming the immunomodulatory effect of β-glucan in a short timeframe [13]. A study by Chiu et al. [80] shows that four weeks of rearing grouper (*Epinephelus coioides*) with *Saccharomyces cerevisiae*-enriched feed resulted in a weight gain of over 200%, compared to the unsupplemented group. Additionally, immunological parameters, including lysozyme activity in the blood, were significantly higher (approximately three times) than in the control group, after 28 days. Fu et al. [81] used a 33-day rearing period of sea bass (*Lateolabrax maculatus*) with probiotic supplementation administered in water. It has been observed that a 30-day rearing period is enough to significantly improve growth and immunity. The supplemented groups showed significantly higher activities of superoxide dismutase, catalase and lysozyme. In addition, changes in the microbiome (increase in the number of microorganisms) were observed. Another study investigated the effect of a feed additive consisting of chitosan and *Acinetobacter* KU011TH on a hybrid of catfish (*C. gariepinus* × *Clarias macrocephalus*) over a 28-day period. Four weeks of rearing were sufficient to demonstrate significant changes in lysozyme activity in the blood, and other immunological parameters, compared to the control group [82]. The results obtained in this study also confirm that a 30-day administration of sodium butyrate, β-glucan and vitamin is sufficient to observe significant changes in the intestinal microbiome and other measured parameters. This is particularly useful in terms of commercialization and the creation of preparations that can be used for a month, thus obtaining good-quality stocking material for further commercial fish breeding.

## 5. Conclusions

The research results presented in this study allow us to conclude the appropriate selection of active ingredients (sodium butyrate, β-glucan and vitamins) in the nutrition of juvenile hybrid sturgeon for the modulation of the intestinal microbiome profile, promoting probiotic bacteria and, at the same time, displacing potentially pathogenic species. Moreover, the above substances positively regulate the immune response of the fish’s immune system. Supplementation increases the activity of lysozyme in the blood—the first line of defense against pathogens. However, compared to the control group, there was no significant reduction in the level of cortisol—the stress hormone—in the supplemented groups. The supplementation also has a positive effect on the growth rate of the tested fish.

Summarizing the obtained research results, it can be concluded that supplementing the diet of hybrid sturgeon with sodium butyrate and β-glucan may increase the efficiency of breeding this species by reducing the occurrence of infectious disease outbreaks. The presented supplementation can become a starting point for creating a complementary feed mixture recommended for use in sturgeon aquaculture.

## Figures and Tables

**Table 1:** Active ingredients of the enriched feed used during the experiment on juvenile sturgeon hybrid (*Acipenser gueldenstaedtii* × *Acipenser baerii*). C–control group, FQV1, FQV2, and FQV3 groups fed enriched feed with sodium butyrate, β-glucan and vitamins.

Active Ingredients *	Groups			
	C	FQV1	FQV2	FQV3
sodium butyrate (mg/kg)	Not included	50	150	50
β-glucan (mg/kg)	Not included	20	20	60
vitamin C (mg/kg)	Not included	30	30	30
vitamin E (mg/kg)	Not included	10	10	10
vitamin K (mg/kg)	Not included	0.4	0.4	0.4
vitamin A (IU/kg)	7500	8700	8700	8700
vitamin D3 (IU/kg)	1125	1925	1925	1925

* The values of vitamin A and vitamin D3 were taken from the feed manufacturer’s label and calculated based on the amounts of added active ingredients.

**Table 2:** Breeding indicators (mean ± SD) of juvenile sturgeon hybrid (*Acipenser gueldenstaedtii* × *Acipenser baerii*) fed commercial feed in the control group (C) and enriched feed with sodium butyrate, β-glucan and vitamins in research groups (FQV1, FQV2,and FQV3) during a feeding experiment. Mean values in the same row marked with a different letter are statistically different (*p* < 0.05).

Breeding Indicators	Groups			
	C	FQV1	FQV2	FQV3
IW (g)	8.56 ± 1.50	8.56 ± 1.50	8.56 ± 1.50	8.56 ± 1.50
IL (cm)	12.50 ± 0.68	12.50 ± 0.68	12.50 ± 0.68	12.50 ± 0.68
FW (g)	49.63 ± 7.53 ^b^	51.68 ± 5.23 ^ab^	54.14 ± 11.16 ^a^	47.20 ± 8.27 ^b^
FL (cm)	22.37 ± 1.15 ^ab^	22.55 ± 0.95 ^ab^	22.91 ± 1.35 ^a^	22.05 ± 1.04 ^b^
WG (g)	40.62 ± 5.64 ^b^	44.04 ± 4.67 ^ab^	47.24 ± 10.54 ^a^	39.66 ± 8.68 ^b^
LG (cm)	9.89 ± 0.95 ^ab^	10.21 ± 0.88 ^ab^	10.57 ± 1.31 ^a^	9.76 ± 0.98 ^b^
SGR (%/day)	4.56 ± 0.52 ^ab^	4.71 ± 0.34 ^ab^	4.82 ± 0.67 ^a^	4.38 ± 0.57 ^b^
K (K/fish)	0.44 ± 0.03	0.45 ± 0.03	0.45 ± 0.02	0.43 ± 0.05
SR (%)	100.0 ± 0.0	100.0 ± 0.0	100.0 ± 0.0	97.2 ± 4.8

IW—initial weight; IL—initial length; FW—final weight; FL—final length; WG—weight gain; LG—length gain; SGR—specific growth rate; K—condition factor; SR—survival rate.

**Table 3:** Data (mean ± SD) from bioinformatic analysis of the intestinal microbiome of juvenile sturgeon hybrid (*Acipenser gueldenstaedtii* × *Acipenser baerii*) fed commercial feed in the control group (C) and enriched feed with sodium butyrate, β-glucan and vitamins in research groups (FQV1, FQV2,and FQV3) during a feeding experiment.

Group	Counts	Observations
C	94,505.20 ± 17,161.98	179.40 ± 87.45
FQV1	105,357.20 ± 12,016.35	254.80 ± 46.91
FQV2	107,145.60 ± 37,173.80	168.40 ± 73.19
FQV3	94,608.00 ± 27,899.12	170.60 ± 66.45
Total number of counts taxonomically classified		2,008,080.00
Mean number of counts per sample		100,404.00 ± 23,546.31
Total number of ASV		1785.00

## Data Availability

The data used to support the results of this study are available from the corresponding author, upon request.

## Supplementary Materials

The following supporting information can be downloaded at: https://www.mdpi.com/article/10.3390/genes15101276/s1, Table S1: Composition of commercial feed used in the feeding experiment on juvenile sturgeon hybrid (*Acipenser gueldenstaedtii* × *Acipenser baerii*).
