# Supplementary material for: Next-Generation Sequencing to Determine Changes in the Intestinal Microbiome of Juvenile Sturgeon Hybrid (Acipenser gueldenstaedtii♀ × Acipenser baerii♂) Resulting from Sodium Butyrate, Β-Glucan and Vitamin Supplementation
Source: Genes (Basel). 2024 Sep 28;15(10):1276. doi: 10.3390/genes15101276 (PMC11507316; doi:10.3390/genes15101276)
Supplement: Supplementary file 1 [file genes-15-01276-s001.zip › genes-3203271-supplementary.pdf]

Table S1. Composition of commercial feed used in the feeding experiment on juvenile sturgeon hybrid (*Acipenser gueldenstaedtii x Acipenser baerii*).

**Composition*:** Fish meal, fields beans, fish oil, soybean meal, wheat gluten, soy protein concentrate, canola oil	
**Ingredients***	**Content ***
crude protein (%)	49
crude fat (%)	19
crude fiber (%)	1.6
raw ash (%)	9.5
calcium (%)	2
phosphorus (%)	1.2
sodium (%)	0.6
Vitamin A (U/kg)	7500
Vitamin D3 (U/kg)	1125
Iron (iron sulfate monohydrate) (mg/kg)	60
Iodine (calcium iodate anhydrous) (mg/kg)	3
Copper (copper sulfate pentahydrate) (mg/kg)	8
Manganese (manganese sulfate monohydrate) (mg/kg)	23
Zinc (zinc sulfate monohydrate) (mg/kg)	130
Zinc (Zinc-amino acid helate) (mg/kg)	50
BHT (antioxidant) (mg/kg)	75

* Values taken from the feed manufacturer's label.
